# Successful management of biliary ascariasis: Case of obstructive jaundice in endemic region – Case report

**DOI:** 10.1016/j.idcr.2025.e02320

**Published:** 2025-07-12

**Authors:** Ebenezer, Gezahegn Fanta, Andrew, Sin Heng Chew

**Affiliations:** a Department: Surgical Department, Myungsung Comprehensive and Specialized Hospital and Myungsung Medical College, Addis Ababa, Ethiopia; b Department: College of Medicine and Public Health, Flinders University, AdelaideAdelaide, South Australia, Australia

**Keywords:** Ascariasis, Biliary system, Round worm, Biliary ascariasis

## Abstract

Ascariasis (round worm) is the largest worm of the common nematodes that infect humans and causes the disease ascariasis. It is most common in tropical and subtropical countries where poor sanitation and illiteracy prevail. Most patients with the disease are asymptomatic; however significant morbidity can arise from the migration of the worm from the small bowel to ectopic sites causing complications. One of the complications is biliary system infestation where the worm travels to the biliary tract.Presentation of symptoms can range from biliary colic to more complicated symptoms such as acute pancreatitis and liver abscess. Early diagnosis and intervention are vital to halt the progression to life-threatening complications. This case report highlights a rare complication of Ascaris infection of the biliary system.

## Introduction

Soil-transmitted helminth (STH) infections are among the most common infections worldwide. It is estimated to affect approximately one-third of the global population [1]. It is widespread among the poorest and marginalized communities in Sub-Saharan Africa, Asia, and South America, where clean water access is limited, and sanitation and hygiene are generally lacking [1], [2]. Ascariasis is one of the STH infections caused by *Ascaris lumbricoides* (round worm). Soil contamination with human feces and utilization of untreated human feces for fertilizer are among the main reasons for the transmission of the disease [1].

Ethiopia is one of the African nations where the prevalence of STH diseases, particularly ascariasis (roundworm), is high. In 2022, about 27 million children required preventive chemotherapy to control the spread of the disease [1]. The introduction of infective eggs of *Ascaris lumbricoides* through contaminated food and water initiates the disease process. The small intestine is the main site for harboring the parasites [2]. Complications like intestinal obstruction and, in some instances, perforation may develop from heavy infestation of the intestine. Other complications arise from ectopic migration of the parasites from the primary site to different parts of the body, such as the hepato-biliary system, urinary system, and lungs. Thus, the disease might have various manifestations depending on the organ system it is affecting [2]. If Ascaris larvae migrate to the lungs, they can cause issues such as pneumonitis, and in rare situations, this can progress to respiratory failure [1], [2].

Biliary ascariasis is one of the common presentations of ectopic migration of the parasite to the biliary system, caused by the migration of the Ascaris from the small intestine to the duodenum and through the Ampulla of Vater, into the biliary system. Relocation of the parasite to the biliary system can lead to complications ranging from mild to severe. This can result in significant morbidity and mortality if not addressed appropriately [2].

This article includes a report on uncomplicated biliary ascariasis managed conservatively and a comprehensive literature review of symptoms, diagnosis, and management.

## Case report

A 30-year-old man visited the emergency department of Soddo Christian Hospital (SCH), in the southern part of Ethiopia. He presented with intermittent non-radiating colicky abdominal pain of two weeks duration. The pain was associated with nausea but not vomiting. He also reported yellowish discoloration of the eye, yellow-tinted urine, and generalized pruritus. He did not report associated fever and denied having undergone any previous biliary system procedure. On examination, his vital signs were in the normal range. He had icteric sclera and a slight palpable rounded mass over the right hypochondrium, with direct tenderness of the upper abdomen; more on the right side. His laboratory investigations revealed a normal blood counts, Erythrocyte Sedimentation Rate (ESR), and renal function tests. Liver function tests were elevated more than 3–4 times above the normal range: SGOT was 317(↑), SGPT 502(↑), ALP 1764(↑), Total Bilirubin 9.4(↑) and Direct Bilirubin 4.62(↑), Table 1. Stool examination revealed ova of *Ascaris lumbricoides* and trophozoite of *Entameba histolytica.*

**Table 1:** Liver function test of the patient during his stay in the hospital. NA: Not assessed.

Liver Function Tests	Admission Days			
	Day 1	Day 2	Day 4	Day 6 (Discharge day)
SGOT	317	83	49	40
SGPT	502	228	115	91
ALP	1764	1432	NA	NA
Bilirubin				
Total	9.4	NA	3.09	2.21
Direct	4.62	NA	1.99	0.92

**Fig. 1:**
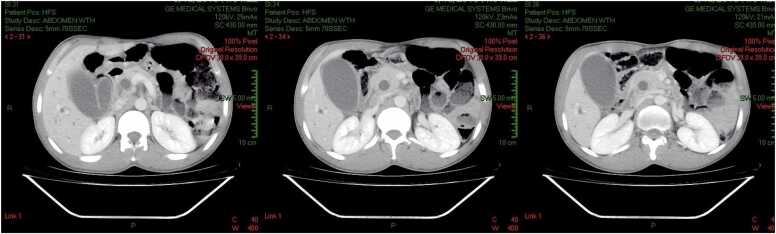
CT Scan: Distended GBD, CBD, and Continuous Hyper-dense structure with central hypo-density (Slice 39–44).

**Fig. 2:**
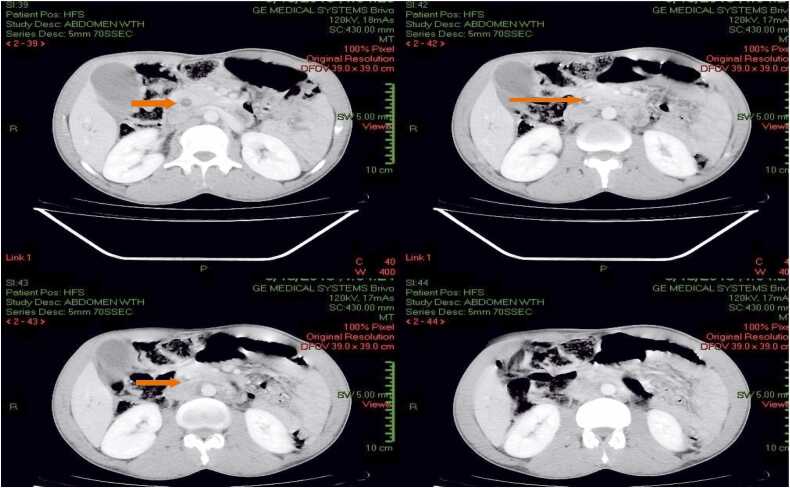
CT Scan: Distended GBD, CBD, and Continuous Hyper-dense structure with central hypo-density (Slice 39–44). The red arrow indicating the filling defect within the CBD.

Abdominal ultrasonography showed intra-hepatic and extra-hepatic bile duct dilation, with the common bile duct measuring 2 cm, and distension of the gallbladder with no identifiable etiology. Gallstones were not detected. CT scan showed marked distention of the biliary ducts and the gallbladder (Fig. 1, Fig. 2). In addition to dilatation of the biliary system, a 2 cm x 5 mm elongated high-density lesion with central hypo-density was found in the distal common bile duct, which was thought to be a parasitic lesion (Fig. 1, Fig. 2).

The patient was presumptively diagnosed with obstructive jaundice secondary to ascariasis based on the clinical presentation of the patient and investigations. He was admitted and treatment initiated. He received intravenous fluids (Normal Saline), metoclopramide injections, omeprazole injections, and ceftriaxone 1 gm intravenous (IV) twice a day, and metronidazole 500 mg IV three times a day (a lower dose was considered due to the absence of signs indicating severe infection). He was also dewormed with albendazole 400 mg. Medical management was pursued for this patient as ERCP was not available within the institution.

The patient improved symptomatically on initiation of treatment, with reduction of his abdominal pain and tenderness. His markers of liver function improved, with parameters becoming near normal at point of discharge on the 6th day of admission (Table 1).

**Fig. 3:**
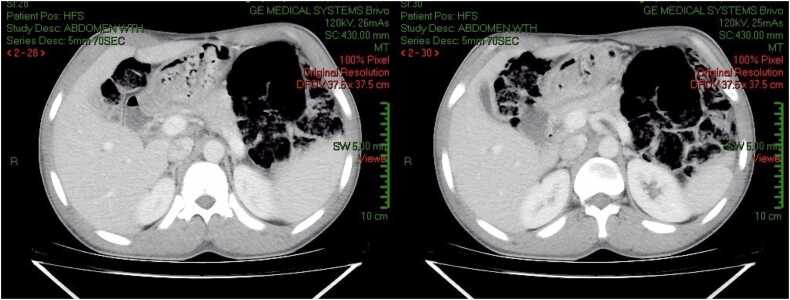
CT Scan: Marked intra and extra-hepatic biliary duct dilation improvements with slight residual dilation. No visible ductal lesions (Slice 28,30).

**Fig. 4:**
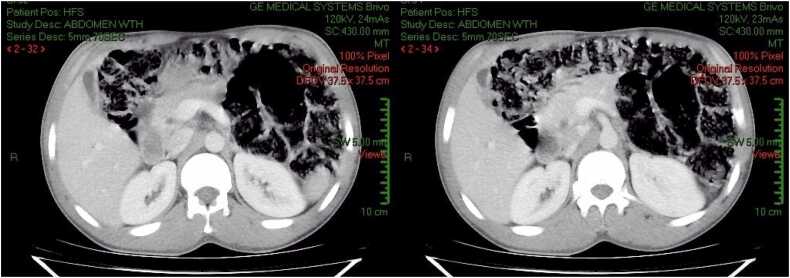
CT Scan: Marked improvement of intra and extra-hepatic biliary duct dilation with slight residual dilation. No visible ductal lesions (Slice 32,34).

Re-imaging with ultrasound and CT scan five days after initiation of treatment showed near complete resolution of biliary obstruction with mild biliary tract dilation and disappearance of the filing defects (Fig. 3, Fig. 4).

The patient was discharged with amoxicillin clavulanate 625 mg and metronidazole 500 mg to be taken three times per day. On followup a week after discharge, the patient remained asymptomatic and no complications were detected. Long-term complications assessment was not possible because the patient was lost from follow-up.

## Discussion

Ascariasis remains one of the most common parasitic infections affecting people with poor living conditions in the tropics and subtropics. This has posed a significant health challenge for these nations [2]. Biliary ascariasis is prevalent in regions with a high load of ascariasis with reporting ranging from 36.7 % in Kashmir to 20 % in the Philippines to less than 1 % in Bangladesh [4]*.*

A significant proportion of patients with biliary ascariasis have a prior history of cholecystectomy [3]. Following cholecystectomy, dilation of the CBD and relaxation of the sphincter of Oddi likely contribute to worm migration into the biliary tree. The same rationale applies to sphincterotomy as well as other procedures performed within the biliary system. This has been shown to play a role in recurrent worm invasion of ducts of people living in endemic areas [3], [4]. The increased prevalence in endemic regions might be due to the rising number of gallbladder and biliary procedures performed on top of the rising poverty, overcrowding, and contamination of water supplies [2], [4].

Despite the lack of sufficient studies, data derived from available limited clinical investigations indicate higher prevalence in women. Although intestinal ascariasis is prevalent among children, the incidence of biliary ascariasis is lower compared to adults [2], [4]. The plausible rationale behind this could be the narrow biliary duct in children that prevents the worms from traversing the papilla of Vater.

The majority of infested people do not have symptoms or present with mild non-specific symptoms. Patients could present with symptoms of ectopic site infestation, leading to hospital admission for further interventions [2]. Biliary ascariasis often presents as a case of biliary colic. Symptoms of cholangitis, cholecystitis, and, less commonly, pancreatitis and hepatic abscess are possible [4], [6]. Of all hospital admissions related to ascariasis complications, biliary ascariasis accounts for about 20 %, even though some reported more [3]. The presence of complications can be one strong indication of high parasitic load, necessitating mass interventions to address the disease burden [2]. Although often overlooked, it is essential to consider ascariasis as a differential diagnosis in patients who present with jaundice and abdominal pain in endemic regions since secondary infection can quickly progress and evolve into life-threatening complications [4]. In our case, the patient presented with biliary colic and jaundice.

In patients presenting with obstructive jaundice and right upper quadrant pain, other infectious causes such as amoebic liver abscess, hydatid disease, and cholangitis secondary bacterial infection should be considered in underdeveloped regions since it is common to observe such cases. It is most important to perform stool microscopy, liver function tests, and imaging studies to aid in narrowing the diagnosis. In this case, the presence of *Ascaris lumbricoides* in stool and a filling defect in the biliary tree on CT scan supported the diagnosis of biliary ascariasis [2], [3], [4]*.*

Ultrasound is an excellent diagnostic tool for visualizing the biliary ductal system. It is safe, available, and noninvasive [5]. However, experience and a high index of suspicion are needed to detect Ascariasis in the biliary system [4]. In the study by Khuroo et al., ultrasound was able to detect worms in the biliary tree in 92.3 % of cases [3], [4]. In this case report, ultrasound was able to detect a dilated biliary system but was not successful in identifying the etiology. Identifying the content of the distal CBD on ultrasound could be anatomically challenging due to the overshadowing of bowel gas from the duodenum. CT scan is an alternative investigation modality that can add information as well. CT evaluates the biliary tree, identifying the possible etiologies in the yielding level [2]. In the presented case, the CT scan was able to detect the biliary tree dilation and the distally located worm in the CBD. Endoscopic retrograde cholangiopancreatography (ERCP) is essential for delineating the biliary tree and identifying the disease. ERCP has the advantage of both diagnostic and therapeutic intervention. It provides endoscopic worm extraction from the bile ducts and further understanding of the biliary tree within the duct [7]. Our hospital did not have such a facility for intervention.

There are two thoughts on management of biliary ascariasis: medical and interventional. When the roundworm migrates from the intestine into the biliary tree, it can carry bacterial flora, which could potentially lead to a secondary infection. Indeed, an infectious complication of biliary ascariasis needs urgent medical and surgical interventions; medical treatment with oral anti-helminth medication and antibiotics supplemented by intravenous fluids are sufficient in uncomplicated biliary ascariasis, though there is a possibility of leaving dead parasites in the biliary system due to paralysis of the worm [2], [3], [6], [7], [8]. Unresolved symptoms might suggest retention of dead parasites in the biliary system, in which scenario endoscopic or surgical clearing of the biliary system is necessary. The other thought of management is early surgical intervention with either ERCP or surgical sphincterotomy and drainage before the initiation of medical treatment to reduce the complications related to deceased parasites in the biliary system. This intervention is more invasive and predisposes for recurrence of the disease in endemic regions where frequent invasion of the biliary system is a possibility due to sphincterotomy. Additionally, the limited availability of ERCP makes it difficult to use in rural hospitals. In our scenario, the patient improved after the anti-helminth medication and antibiotics. The chosen antibiotics were intended to provide coverage for possible bacterial contamination of the biliary system, including anaerobic organisms despite the absence of fever suggestive of ascending cholangitis. Metronidazole was included in the treatment regimen additionally to address the *Entamoeba histolytica* identified in stool examination, although luminal agents such as paromomycin or diloxanide would have been more appropriate if they had been available.

Given the limited access to ERCP at our facility, surgical intervention—specifically open common bile duct exploration—was considered as a plan in case of clinical deterioration or persistent parasitic presence in the bile duct despite medical therapy. It is not unusual for the parasite to migrate back out of the biliary tree to the intestine, decompressing the biliary system [2], [3], [8]. Our patient did not require invasive procedures or develop other complications.

## Conclusion

Intestinal parasitic infections like ascariasis impose a significant burden on healthcare systems in developing nations. Addressing the issue will reduce the disease burden and its complications. It is crucial to consider ascariasis as a differential of biliary disease in endemic regions. Early diagnosis and immediate measures should be taken to treat biliary ascariasis. Conservative management avoids invasive interventions required to remove the worm from the duct. On need of invasive interventions, the availability of ERCP reduces the invasiveness of the procedure.

## Ethical clearance

Permission for publication of this case-report was obtained from Soddo Christian Hospital and informed consent was obtained from the patient.

## Funding

No source of funding.

## Declaration of Competing Interest

The authors declare that they have no known competing financial interests or personal relationships that could have appeared to influence the work reported in this paper
